# Biped Gait Stability Classification Based on the Predicted Step Viability

**DOI:** 10.3390/biomimetics9050265

**Published:** 2024-04-27

**Authors:** Pedro Parik-Americano, Jorge Igual, Larissa Driemeier, Eric Cito Becman, Arturo Forner-Cordero

**Affiliations:** 1 Biomechatronics Laboratory Mechatronics Department, Escola Politécnica, University of São Paulo (EP-USP), São Paulo 05508-030, Brazil; pedro.americano@usp.br (P.P.-A.); driemeie@usp.br (L.D.); eric.becman@usp.br (E.C.B.); aforner@usp.br (A.F.-C.); 2 Departamento de Comunicaciones, Instituto de Telecomunicaciones y Aplicaciones Multimedia (ITEAM), Universitat Politècnica de València, 46022 Valencia, Spain

**Keywords:** biped stability, classification, real-time application, predicted step viability

## Abstract

In this paper, we address the challenge of ensuring stability in bipedal walking robots and exoskeletons. We explore the feasibility of real-time implementation for the Predicted Step Viability algorithm (PSV), a complex multi-step optimization criterion for planning future steps in bipedal gait. To overcome the high computational cost of the PSV algorithm, we performed an analysis using 11 classification algorithms and a stacking strategy to predict if a step will be stable or not. We generated three datasets of increasing complexity through PSV simulations to evaluate the classification performance. Among the classifiers, k Nearest Neighbors, Support Vector Machine with Radial Basis Function Kernel, Decision Tree, and Random Forest exhibited superior performance. Multi-Layer Perceptron also consistently performed well, while linear-based algorithms showed lower performance. Importantly, the use of stacking did not significantly improve performance. Our results suggest that the feature vector applied with this approach is applicable across various robotic models and datasets, provided that training data is balanced and sufficient points are used. Notably, by leveraging classifiers, we achieved rapid computation of results in less than 1 ms, with minimal computational cost.

## 1. Introduction

The robotics community has always been fascinated by biped walkers. Their humanoid form and ability to locomote on human-centered environments have sparked interest into their potential applications. For instance, bipedal robots can replace humans in hazardous situations, such as landmine-ridden fields or radioactive zones. Or consider the possibility of exoskeletons aiding human mobility when compromised by neuromuscular diseases.

However, one of the major challenges in designing bipedal robot walkers is the definition of criteria to generate stable gait trajectories guaranteeing that the robot does not fall during gait. In humans, a combination of neuromechanical factors results in stable, robust, versatile, and energy efficient gait [1,2]. However, since the state of the art of motor control neurophysiology cannot fully model the complete human stability control during walking, the biomimetic translation of these characteristics into biped robots is not an easy task. Moreover, the dynamics of biped locomotion is nonlinear, due to its strong dependency on the system configuration [3], and thus trajectory planning and execution to ensure stability is a complex problem.

In 1969, Vukobratovic et al. introduced one of the first stability criteria, the Zero Moment Point (ZMP), for biped robot walking pattern generation and control [4]. The ZMP is the point on the ground where the resulting torque of inertial and gravitational forces on the robot has no horizontal component. This requires the resulting forces between the feet and the ground to be located within the region defined by the contact between the feet and the ground.

Although this approach allowed the development of many biped walking robots, the constraints imposed by the criterion result in some drawbacks on the robot movement. The need to position the center of pressure (CoP) inside the support polygon implies that the robot cannot fully exploit the inverse pendulum dynamics. This results in high energy consumption with a slow and unnatural motion of the robot compared to human walking. Furthermore, it requires high accuracy in the measurement of the robot joint positions.

Several techniques have been proposed to overcome these limitations. For instance, in [5] the authors extended the ZMP introducing the Preview Control theory to achieve a stable gait adapting to uneven terrains. The step capturability criterion (SC), proposed by Pratt and Tedrake [6], is based on the definition of a point on the ground for foot placement, in such a way that, reaching this point, the robot can stop at static equilibrium at all joints: the capture point. This idea has a clear counterpart in the human gait [7], and it also related to the mechanism employed by humans to recover from a trip [8]. In 2012, Koolen et al. [9] expanded the SC criterion to the N-Step Capturability (N-SC), in such a way that it is possible to reach the capture point in a certain number of steps.

Later, the predicted step viability (PSV) was proposed in [10], inspired by the N-SC idea and the human ability to recover from perturbations, such as tripping. In this way, a gait is considered stable if the biped is able to reach a capture point in a finite time. It reduces the constraints imposed to the current step in such a way that it only has to end in a configuration that future steps are able to bring the robot to a capture point.

To determine the capability of the biped to reach a capture point, the PSV has to plan the desired gait pattern and verify whether this pattern satisfies the stability criterion at the beginning of each step. This is achieved via a multiphase optimization proble as it is based on the predicted behavior of future steps. Using this criterion, the gait pattern can be non-cyclic as the human gait while walking on irregular surfaces. Given some desired gait parameters such as step length, center of mass (CoM), height, horizontal velocity, and trunk inclination, the algorithm optimizes the step to get as close to these parameters as possible while reducing the capture point distance and adjusting other parameters such as the step duration, advancing or delaying the foot contact as needed. This means that the algorithm can self-adapt to maintain critical constraints but ensure fast recoverability as it is found in the human gait when recovering from a trip [8]. The major limitation of the PSV is the complexity of the optimization to plan each step. Since the algorithm uses the complete robot dynamics to assess the recoverability of each possible step, it cannot be directly applied to control a biped robot in real-time.

In summary, the PSV is a powerful stability and trajectory planning criterion that optimizes joint trajectories to minimize consumption and maximize stability, ensuring the existence of subsequent stable steps. However, in its analytic formulation, it is impossible to apply to real-time embedded systems. In this paper, we explore machine-learning-based solutions that are able to implement the PSV stability criterion in real time, i.e., classify whether the step is going to be stable or not. We will analyze a set of classifiers coming from different classification paradigms. We will test and compare them in 5-segment biped walkers, such as robots and exoskeletons.

## 2. Materials and Methods

### 2.1. Predicted Step Viability

**Figure 1:**
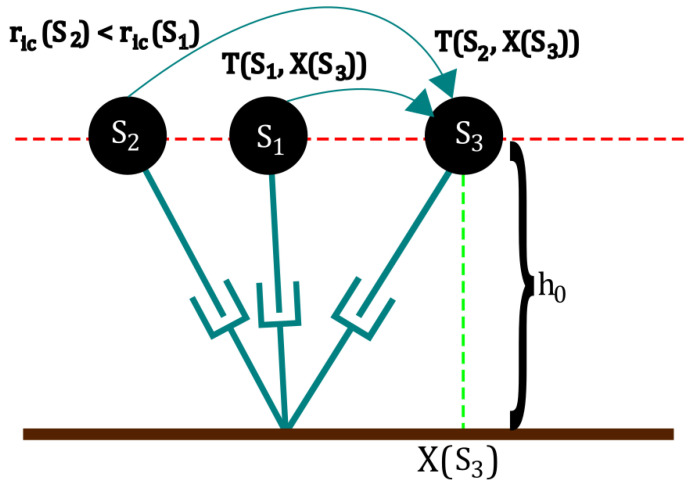
Visual representation of the PSV criterion. Source: adapted from [10].

In the PSV, the current step is planned to guarantee that the next step exists on which the robot is capable of either reducing or maintaining the capture point distance [10]. This multistage optimization problem guarantees the stability of the robot and the viability of subsequent steps, along with minimizing energy consumption. Figure 1 shows a visual explanation of the basis of the PSV criterion.

Given an initial distance to the capture point $r_{ic}\left(S_{1}\right)$, on which $S_{1}$ is the set of initial conditions (positions and velocities) of the robot, and a fixed set of actuations $U_{1}$, the robot will drive itself to a given configuration $S_{3}$, defined by a fixed distance from the support point $X(S_{3})$, in a given time $T(S_{1},X\left(S_{3}\right))$. This movement is ruled mostly by the exponential diversion of the capture point and thus the passive joint. In this situation, for a second, closer capture point position $r_{ic}\left(S_{2}\right)$, the robot will have a time $T\left(S_{2},X\left(S_{3}\right)\right)>T\left(S_{1},X\left(S_{3}\right)\right)$ to drive itself to the same configuration $S_{3}$ using an actuation $U_{2}\leq U_{1}$. Consequently, if the robot can perform the same step, using a higher actuation $U_{2}<U_{3}\leq U_{MAX}$, we will have $t_{3}<t_{2}$ and consequently finish the step with a capture point closer than with the previous actuation.

If a possible step exists that reduces the capture point distance in the next step, then the following step that will further reduce it will also exist. Therefore, the robot can eventually bring itself to a full stop, in the absence of external disturbances. Conversely, if there is no possible step that can at least maintain the current distance to the capture point, then a fall is inevitable.

The PSV is a powerful stability and trajectory planning algorithm that guarantees the stability of the current step and the existence of all subsequent steps. Simultaneously, the PSV also aims at enhancing the step feasibility by optimizing a cost function that includes the maximum joint torques, the trunk inclination, and the maximum step length. Notably, other parameters are not needed, such as stepping time, which allows anticipation or delay in contact with the floor as needed to guarantee stability. This maintains the generality of the method while increasing the robustness to external disturbances. However, the main drawback of the method lies in the multistage optimization problem that must be solved to plan the trajectory and check the recoverability of the current step, thus rendering its application in real time impossible with current technology.

### 2.2. Robot Model

In this work, two different biped robot models were used to assess the capability of the proposed method to work with different bipeds and the generalizability of each set of trained classifiers to work with different models. The parameters of each biped can be seen in Table 1 and Table 2. While the first represents an adult-size full body exoskeleton, the second represents the parameters of a small-sized robot.

**Figure 2:**
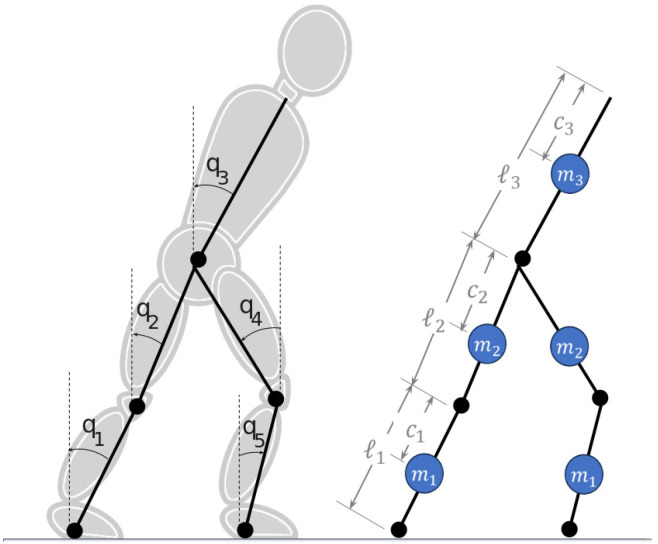
Planar 5-segment robot based on RABBIT.

Both models are modified versions of the classic 5-segment robot with point feet RABBIT [11]. They consist of five segments with known center-of-mass positions. The angles are defined with respect to the vertical as shown in Figure 2. All signal conventions follow the trigonometric circle convention. Motion is limited to the sagital plane, and thus there is no collision between parts of the robot. Also, the feet, as in the original RABBIT model, are punctual and have a collision box with the ground. Moreover, the robot is modular, with the mass, length, CoM position, and inertia of segments 1 and 2 being equal to 4 and 5. Finally, segment 3 models the torso of the robot, which contains most of the mass and thus governs the CoM position.

The PSV method was implemented as described by Rossi et al. in [10], and a set of simulations were performed to map the initial and end-step conditions of the biped for each initial configuration. The result of the algorithm, which means whether or not the robot managed to reduce the distance to the capture point (i.e., the step is recoverable), is also registered for posterior analysis.

The set of all possible initial conditions for the robot are defined with three constraints. First, the front leg and the torso must not initially be bent backward to prevent a step back from the robot. For the same reason, all joint velocities must be negative according to the angle convention presented in Figure 2. Second, the internal angles between the robots thighs and legs must be positive but smaller than π (i.e., the leg cannot be bent forward). And third, both feet must be touching the ground at the beginning of the step and the swing foot starting position must be behind the support foot. They are formulated mathematically using the following equations:

$q_{1}\in ]-\left(\pi /2\right);0]$ and ${\dot{q}}_{1}<0$

$q_{2}\in ]q_{1};\pi +q_{1}]$ and ${\dot{q}}_{2}<0$

$q_{3}\in ]-\left(\pi /2\right);0]$ and ${\dot{q}}_{3}<0$

$q_{4}\in ]\arccos\left(\frac{l_{1}\cos\left(q_{1}\right)+l_{2}\cos\left(q_{2}\right)}{l_{1}+l_{2}}\right);q_{2}]$ and ${\dot{q}}_{4}<0$

$q_{5}=\arccos\left(\frac{l_{1}\cos\left(q_{1}\right)+l_{2}\cos\left(q_{2}\right)-l_{2}\cos\left(q_{4}\right)}{l_{1}}\right)$ and ${\dot{q}}_{5}<0$

### 2.3. Datasets

Three increasingly difficult data sets were generated to assess the viability of the approach and to test the robustness and generalization of each classifier.

The first dataset presents an exploration of the complete set of joint angles for the robot while keeping the initial velocity for each point of the dataset constant for each joint and with a value of −1 rad/s. No constraints on the evolution of joint angles and velocities were imposed other than the mechanical limits of the robot and the maximum torque of the motors. This dataset allows for the exploration of which extreme conditions, which could arise after slipping or external influence, for instance, could still be recoverable. The value of −1 rad/s was chosen as a compromise between the fast and slow movements of the swing and stance legs, respectively. At the same time, we analyzed the theoretical initial n-step capturability of the system for each condition and verified if the chosen value would result in a distribution across all of the theoretically recoverable zones, while minimizing points above $n_{\infty }$. A finer discretization of the workspace is performed around the angles of each segment. The discretization step was set to 0.157 rad. A total of 30.370 different conditions were simulated with the exoskeleton model for this data set, of which 11.4% were recoverable steps.

For the second dataset, both position and velocity were explored. We maintained the same workspace for the position but increased the step to 0.5240 rad. As for velocity, we gradually increased the maximum angular velocity and compared the resulting theoretical n-step capturability of each condition. The maximum velocity was chosen so that most points would fall within the theoretically recoverable zone by the n-step criterion. The chosen range for velocities was from −1 to −10 rad/s, with 2 increments of 4.5 rad/s. However, since the possible configuration of the robot could differ largely from the ideal scenario on which the inverted pendulum model is based, and there was a large excursion of the center of mass both horizontally and vertically, most of the points would result in a fall. A total of 67.068 different conditions were simulated with the robotic model, of which 4.6% were recoverable steps.

These datasets were used to assess the effect of the dataset on the training and the sensitivity to unbalanced data. By analyzing their combined results, we explore how important initial velocity can be for the overall performance of the classifier or if the position mostly dictates the recoverability.

The third dataset was chosen to better represent the operation point of the biped robot. Three constraints were applied to the initial conditions of interest to obtain a balanced dataset, i.e., a dataset with a similar number of recoverable and non-recoverable steps. First, we limit the height of the CoM in the initial configuration to be above 74% of the CoM that the human gait resized to the robot’s parameters would have. Second, we limit the initial vertical velocity of the CoM to be above −0.6 m/s. Third, we calculate the theoretical n-step capturability of each condition and remove all points that would theoretically need more than 3 steps to reach the capture point. Finally, since some of the points were removed due to the 3 rules, we chose a finer step of 0.3140 rads for position exploration and 3 rad/s for velocity exploration. A total of 74.618 different conditions were simulated with the robotic model, of which 44.4% were recoverable steps.

### 2.4. Classification

We trained 11 different classifiers to predict whether a given step falls within either the stability region or the no-recovery zone for each dataset. They are: Naive Bayes (NB), Logistic Regression (LogReg), k-Nearest Neighbors (KNN), Support Vector Machine with Linear Kernel (SVM-L), Support Vector Machine with Radial Basis Function Kernel (SVM-RBF), Decision Tree (DT), Random Forest (RF), Adaboost (ADA), Quadratic Discriminant Analysis (QDA), Linear Discriminant Analysis (LDA), and Multi-Layer Perceptron (MLP). They were selected to explore various classification approaches, spanning from linear to non-linear, parametric to non-parametric, and generative to discriminative solutions [12,13]. In addition, we introduced an ensemble approach that consolidates the outputs generated by each individual classifier. Except for MLP, all of the preceding methods are combined using the stacking technique [14]. Since we are only interested in real-time and simple implementations, we did not consider any deep-learning-based approaches.

The input feature vector $x=[x_{1},\ldots ,x_{15}]$ comprises the positions $x_{1},\ldots ,x_{5}$ and velocities $x_{6},\ldots ,x_{10}$ of five segments illustrated in Figure 2; the center-of-mass (CoM) position vector $x_{11},x_{12}$ and velocity $x_{13},x_{14}$; and the relative position of the capture point concerning the support feet $x_{15}$. This approach indirectly incorporates robot-model-related information without the necessity of including mass or inertia distribution, which would tie the solution to a specific model. Due to the prevalence of unstable conditions stemming from extreme robot configurations, resampling was essential for training the classifiers in datasets 1 and 2. The Synthetic Minority Oversampling Technique (SMOTE) with 9 neighbors was employed to balance these datasets [15].

### 2.5. Model Training and Validation

**Figure 3:**
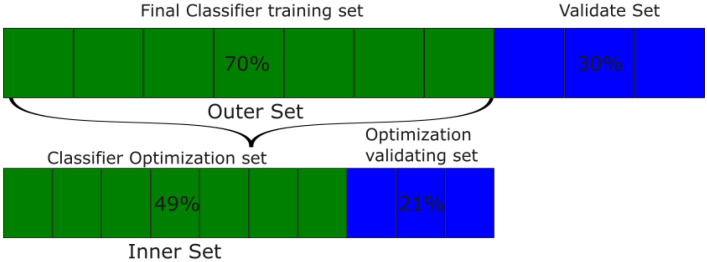
Visual representation of the data division sets used in this work.

The nested holdout validation strategy was implemented to find the optimal hyperparameters of each classifier. In total, 70% of the data (outer set) was set aside for the optimization of the hyperparameters. From this subset, 70% was used to train each classifier with each hyperparameter on a grid search paradigm, while the remaining 30% was used to test each classifier and choose the best one. After finding the best hyperparameters, each classifier is retrained in the totality of the Outer Setand the remaining data are used to compute the final score of each method, referred to as Validate Set. Figure 3 summarizes the data distribution for each stage. Each of the 11 classifiers follows the same procedure.

It is reasonable to expect that, under normal conditions, undisturbed robotic gait will mostly comprise recoverable points. Nonetheless, it is important that the classifiers are able to identify when we have exited this stability zone and should increase gait robustness or prepare for a fall. In this scenario, there is a prevalence distinction on the stable class from training data and real use scenarios. Therefore, the scoring method for the classifiers should be independent from the class prevalence so we do not introduce a bias in our training. In this way, the performance of training and real life should be the same. So, we must be careful in the selection of the metric to be used for the hyperparameter optimization, e.g., in [16] it is explained why the use of f1-score could lead to problems if there is a large difference between the prevalence of stable class on training and application. In our case, we used the balanced accuracy since it is not affected by the prevalence of the classes, so there is not a risk of obtaining a biased classifier in favor of one of the two classes. Once the hyperparameters for the corresponding classifier are optimized, each classifier is run (for each dataset) and different metrics are calculated: balanced accuracy, ROC AUC, f1 score, average precision, precision, recall, specificity, and negative predictive value (NPV) [17], as well as the time taken for a prediction for trained classifiers.

In the biped gait framework, falsely identifying a condition as belonging to the stability region can lead to damage to the device or, in the case of exoskeletons, to the wearer. On the other hand, falsely identifying a condition as part of the no-recovery zone would lead to increased computational costs as the algorithm would try to increase robustness of the gait to recover from instability or prepare to minimize damage in an eventual fall. Since misidentification is expected to occur around the boundary of regions, increasing the robustness of gait on a false negative (FN) can lead to faster recovery and convergence towards a more stable region in a situation that, while still stable, would be approaching an irrecoverable situation. On the other hand, not trying to do so in a false positive (FP) could distance the robot even further from the boundary, resulting in a completely irrecoverable situation and unavoidable fall. For this reason, while both are not ideal and should be avoided, the FP are more problematic.

Finally, an Out-of-Sample evaluation was performed by running the trained classifiers of all datasets on the validating set of each data set and comparing the percentage of TP, TN, FP, and FN within each data set. Since each dataset has different prevalences of positive and negative classes, the percentage of each prediction is given to simplify a direct comparison. This way, we can verify if models hold their performance when applied to datasets other than those on which they were trained on and, thus, if the classifier could be generalized for a broader range of robots or must be model specific.

### 2.6. Robotic Simulation Validation

Finally, to validate the results obtained with the training of the classifiers on the PSV output, a numerical simulation of the robotic model described in Table 2 and used for datasets 2 and 3 was performed. MATLAB version: 9.14.0 (R2023a) (The MathWorks Inc., Natick, MA, USA) was used in this step. At the end of each step, the procedure described in [18] was used to compute the new states of the robot post impact.

A motion planner was implemented as described in [19] to control the robot states. The states considered and that were controlled in this study are:

1. Torso inclination in respect to the vertical (Γ);
2. Vertical position of the center of mass ($CoM_{y}$);
3. Both vertical and horizontal position of the swing foot ($P_{{sw}_{x}},\;P_{{sw}_{y}}$);

Similar to [18], our robot employs Bézier Curves to interpolate and smooth the trajectories for motion planning. The use of Bézier curves allow one to accommodate for initial derivatives at the beginning of the step, thus minimizing required torque, while at the same time having a controlled evolution to the desired end condition. In this study, the torso reference was kept vertical, and CoM height was kept constant at a height equivalent to the human CoM resized to the robot’s dimensions. Swing foot height initially follows the derivative resulting from the impact dynamics and is then kept at a constant height before being projected to the ground. Finally, on each iteration of the control loop, the diversion of the capture point position at the end of the step is estimated, based on its current position and the planned step duration. The Bézier points are then updated based on the new prediction, to smooth the step trajectory at a fixed distance from it.

The simulation was repeated with increasing levels of disturbancies, from model mismatch, sensing noises, terrain irregulaties, and pushes. For model mismatch, a random difference up to 50% was added or subtracted to the robot model but not to the controller model. The mass, inertia, and center of mass positions were varied. Encoder sensing noise was modeled as the sum of two Bernoulli sequences, one positive and one negative, with a probability 99% of being kept at zero, while tachometer noise was modeled as Gaussian noise. Finally, both the push and the terrain inclination were sequentially increased until the control could no longer stabilize the robot, leading to a fall. In all cases, the disturbance was introduced in the 5th step to give the robot time to reach steady state before introducing them and was kept untill the 50th step.

We then proceeded to use the best performing classifiers to predict the recoverability of each step using only the initial conditions of each step.

## 3. Results

### 3.1. Real-Time Performance

One of the motivations of this work is to prove that it is possible to obtain an accurate prediction of the PSV criterion in real time. Although in this study only the stability evaluation of the PSV is being reproduced and not the trajectory planning, all of the proposed methods managed to reduce the computation time of the PSV algorithm significantly, allowing for their real-time use. The neural network classifier requires more time due to the complexity of the model as expected: near 5 ms per prediction when applied on a raspberry pi 3 model B, Raspberry Pi Foundation, Pencoed, Wales, UK. KNN and SVM-RBF took 1 ms amd 800 μs, respectively. For the rest of classifiers, the response is even faster. When it comes to the stacking classifier, the total time to compute an answer was close to the sum of the individual times of the involved methods, taking around 6 ms.

### 3.2. Classification

**Figure 4:**
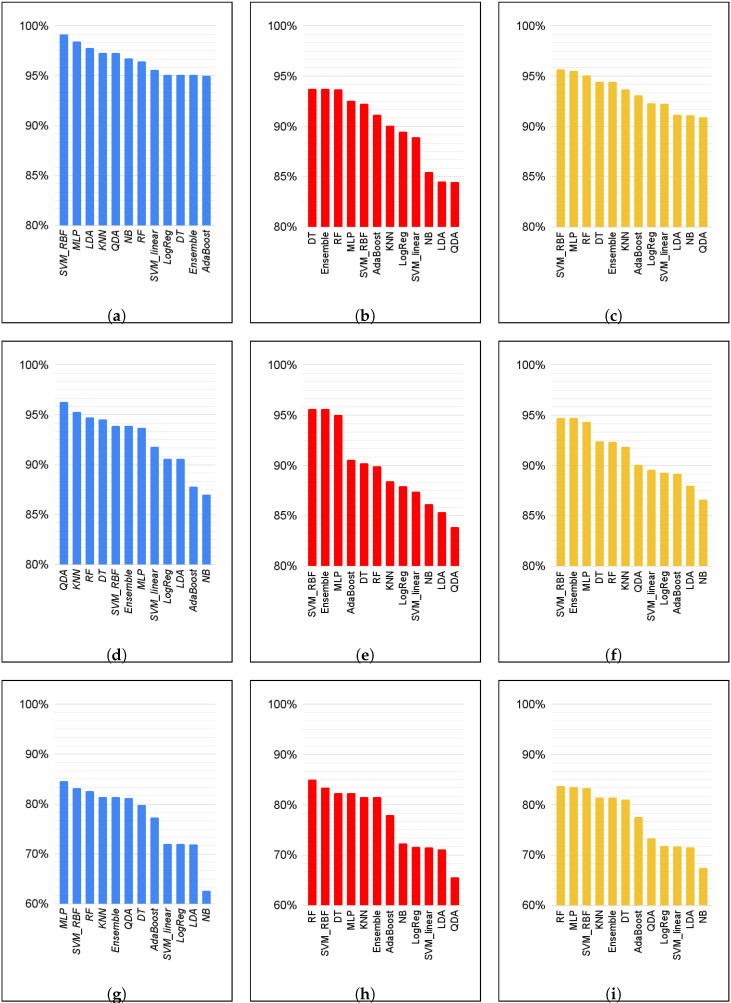
Summary of the three main metrics of all classifiers in all datasets. (**a**) TPR dataset 1. (**b**) TNR dataset 1. (**c**) BAcc dataset 1. (**d**) TNR dataset 2. (**e**) TPR dataset 2. (**f**) BAcc dataset 2. (**g**) TPR dataset 3. (**h**) TNR dataset 3. (**i**) BAcc dataset 3.

**Figure 5:**
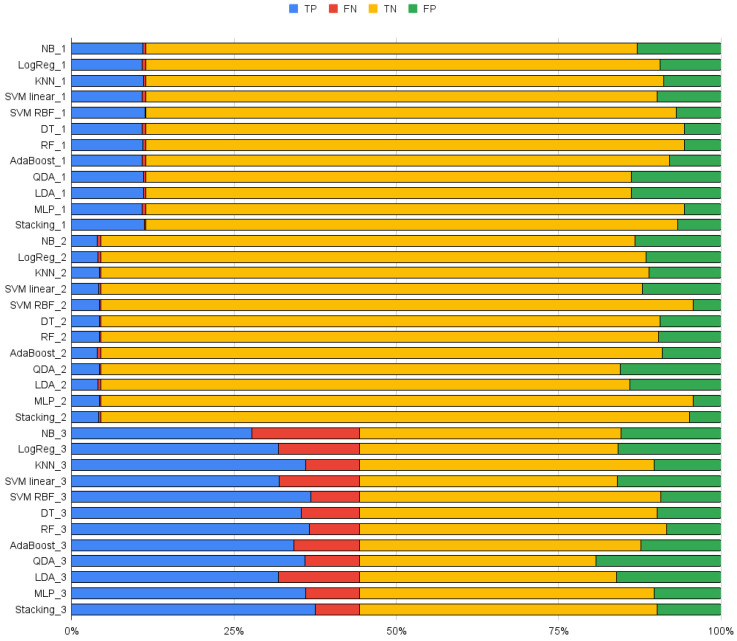
Summary of the results of each classifier for each dataset.

Table 3, Table 4 and Table 5 summarize the results for each classifier for the validation of the set of data in datasets 1, 2 and 3, respectively. For the comparison of the different results, we will analyze the sensitivity, also known as true positive rate (TPR); the specificity, also known as true negative rate (TNR); and the balanced accuracy (BAcc). A visual representation of these metrics for each algorithm and dataset is given in Figure 4. As a visual summary, in Figure 5 we show the overall performance of each classifier.

We can see in Figure 4 that SVM-RBF performs well in all datasets and metrics; it is consistently ranked in the top three classifiers and always at least in the top five. MLP, RF, DT, stacking, and KNN also consistently rank highly. In terms of TNR, DT, RF, and SVM-RBF obtained the best performances in dataset 3; SVM-RBF, stacking, and MLP performed better in dataset 2; and DT, RF, and stacking performed better in dataset 1. Moreover, we can see that there is a clear drop in performance in dataset 3, with the best three classifiers having around 93% TNR in dataset 1, over 95% in dataset 2, and around 84% in dataset 3. Looking exclusively at FP, this translates to around 6%, 4.37%, and 9.14% FP misidentification for datasets 1, 2, and 3, respectively. At the same time, we found that KNN also managed to retain high TNR in all three datasets. When it comes to TPR, SVM-RBF still performed well, scoring 99%, 94%, and 83%, respectively. We found, however, that LDA and QDA performed well in dataset 1 and 2 in this scoring but performed worse for TNR in dataset 3.

While SVM-RBF, MLP, DT, RF, KNN, and the stacking of methods had significantly high balanced accuracies, LDA, QDA, SVM-L, NB, and LogReg had significantly worse performances. In all cases, the TNR of these methods was worse, albeit at times they had high TPR. Balanced Accuracy was consistently worse than the other methods. When it came to dataset 3, the NPV of these methods was also lower, indicating that the algorithm found it difficult to properly separate regions within the data set.

All of the methods performed worse in dataset 3, which had a more focused distribution of points and contained more points around the boundary between stability and no-recovery zones, making the problem much harder. DT and the stacking method had the greatest performance reduction when compared to dataset 1. RF, MLP, and SVM-RBF were the algorithms that were more robust to this dataset, maintaining high balanced accuracy.

### 3.3. Stacking Performance

The use of the stacking strategy to combine the different classifiers did not improve the overall performance. In all cases, the stacking results were worse than the ones obtained with the best single algorithm. Most notably, SVM-RBF outperformed the stacking method for all conditions, except TNR in dataset 1, while having a significant lower prediction average time.

### 3.4. Out-of-Sample Validation

Each trained classifier was tested on all data sets to compare the performance on a data set other than the one it was trained on. Table 6, Table 7 and Table 8 summarize the results.

When it comes to model translation, our results show that there is performance reduction when using a classifier trained on one model on top of another and that it is much greater if there is also a significant difference in data set form. Table 6 and Table 7 show that there is a significant increase in either FP and FN for all classifiers, except when trained with dataset 3.

All classifiers trained in dataset 2 performed worse when applied to the other datasets. Out of the best performing ones, DT and RF saw the least significant performance reduction when applied to dataset 1. These algorithms had 4.7% and 3.9% more FP, and 0.4% and 0.3% more FN, respectively, for a total of 14% and 13.5% FP, or 84.1% and 84.8% TNR, and 0.6% and 0.5% FN, or 94.4% and 95.1% TPR, respectively. However, even if the performance decreases of other methods such as SVM-RBF and stacking were greater, since they had better performances in dataset 2, the overall performance was very similar with 14.3% FP and 0.8% FN for both. KNN and MLP saw a much more significant performance decrease, on the other hand. When applied to dataset 3, however, the performance reduction was much more expresive for all methods, having more than 20% extra FP for all methods.

When trained in dataset 1, only MLP managed to retain performance when tested with data set 2. It showed a small increase in both misidentifications, having 3.8% extra FP and 0.7% extra FN, for 89.1% TNR and 80.4% TPR. When applied to dataset 3, all methods had significant performance reduction.

Finally, when it comes to dataset 3’s trained classifiers, SVM-RBF and MLP performed well on both datasets. However, since the number of true positives in datasets 1 and 2 is smaller than in dataset 3, the small increase in misdentification resulted in a lower TPR. SVM-RBF had a total of 91.3% TNR and 68.9% TPR in dataset 1, and a similar 99% TNR and 61.1% TPR in dataset 2. MLP, on the other hand, had 88.6% TNR and 89.1% TPR in dataset 1, and 99% TNR and 66.5% TPR in dataset 2.

### 3.5. Simulation Results

KNN, DT, RF, and SVM-RBF were used to predict the stability of each step in increasingly unstable conditions. All four classifiers managed to successfully identify that the steps were recoverable in all conditions, with all degrees of disturbances. Terrain inclination varied from −3° (−5.2%) to 20° (36.4%), while a constant push was applied to the robots hip of up to 7N (12% of robot’s mass).

In conclusion, the results obtained for all these experiments prove that it is possible to provide an embeddable real-time classifier that can replace the PSV analytical formulation in a real robot.

## 4. Discussion

Accurately and rapidly identifying unstable conditions that could lead to a fall is critical for ensuring the integrity of biped robots and exoskeletons alike. Machine learning techniques have become increasingly common due to their versatility, accuracy, and speed of prediction, managing to combine information from complex and varied sources to overcome the limitations of simpler models and the computational cost of complete dynamic models. Over the years, different groups have studied how to best combine various sensors to correctly classify the stability of the gait. Solutions have ranged from inertia measurement units on multiple body parts [20], to integrating trunk acceleration and CoP position [21], or using physical quantities derived from the robots’ own sensors [22]. Input features and training data are critical for the success of such techniques. As such, using complex models that can extract more information about the robots’ gait and including both kinetic and dynamic information on the input feature could be the best approach to ensure the generalization and accuracy of ML-based techniques. Despite researchers’ best efforts, some conditions that may arise during walking are simply irrecoverable. For these, other groups have studied how to minimize potential damage to the system [23,24].

In our work, we thoroughly examined different classifiers to assess the viability of implementing the PSV stability criterion in a real-world robotic system under real-time constraints while also focusing on minimizing computational cost and energy consumption. Additionally, we investigated the generalizability of our findings across two biped models and three datasets. This is crucial because maintaining robustness against model parameters is essential for lower limb exoskeletons. While the number of degrees of freedom remains consistent across different users, variables like mass, the segment center of mass positions, and inertia vary significantly. So, a stability classifier can adapt to these variations to ensure safe exoskeleton operation for different users. To test this adaptability, we evaluated the classifiers by training and testing them on a biped system that has different inertial parameters yet with the same number of degrees of freedom.

All the classifiers investigated in this study significantly simplified the PSV algorithm complexity, reducing the prediction time to less than 5 ms, which is adequate for real-time applications. Since robotic systems often operate at sampling frequencies of 1 kHz or higher, it is essential to note that this prediction only needs to run once per step. In this scenario, it is possible to assess stability separately and in parallel with trajectory planning computation and execution.

While the joint control loop could be run at 1 kHz, the step period could range from 400 ms to 1 s depending on the biped robot size. In this case, the 0.8 ms classification delay shown by the SVM-RBF corresponds to less than 0.25% of the stepping time. Furthermore, our simulations demonstrated that the strategy is robust to model mismatch, sensing errors, and light disturbances in both the terrain inclination and pushes, further validating the model’s efficacy. For this reason, SVM-RBF performed better in this criterion as the lower computational time with respect to MLP allows for the computation of the predicted stability of several steps ahead when planing trajectory.

Among the classifiers, SVM-RBF, MLP, RF, DT, and KNN obtained the best results, consistently ranking at the top of the different TPR, TNR, and BAcc metrics. The performance of the joint method of stacking all classifiers was similar to that of the highest performing individual classifiers. Due to its higher computational cost, further analysis ruled out the stacking method.

SVM-RBF and MLP demonstrated higher recall and specificity than the other methods. However, KNN consistently had more mislabeling of FP, as depicted by a lower precision and specificty, despite its high recall and low false negatives. It is also important to note that in this case, false positives FP are much more harmful than false negatives FN. While an FN would induce additional resource use from the robot to increase step robustness (e.g., by reducing center of mass and increasing step length and cadence for a few steps), an FP could result in a fall, possibly damaging hardware or, in the case of exoskeletons, the wearer.

KNN, SVM-RBF, DT, and RF demonstrated excellent performance when dealing with distinct boundaries between classes (recoverable and non-recoverable zones). KNN’s non-parametric nature allows it to thrive on local data distributions, making it effective for well-separated classes. DT and RF excel in partitioning informative features recursively, accommodating non-linear relationships and capturing interactions among features, thereby leveraging clear data separation. Meanwhile, SVM-RBF’s capability to create non-linear decision boundaries by mapping the feature space to higher dimensions becomes advantageous when distinct and well-defined regions exist within the feature space, aligning with our well-separable stable and non-recovery zones. The overall high performance of these four methods indicates a clear separation between classes in our data, suggesting the presence of a basin where the trajectory planning algorithm should focus to maximize gait stability and ensure the recoverability of the next step.

In contrast, LDA and SVM-L seek linear decision boundaries for classification, explaining their poorer performance in classifying non-linear or complex relationships between features and classes. However, MLP’s high flexibility in mapping interrelations between features and classes enables it to handle high-dimensional, non-linear, and complex data by approximating any function mapping inputs to outputs. This flexibility allows MLP to maintain high precision and NPV when other methods fail to do so.

Regarding model adaptation, our findings suggest that it is feasible to maintain or even enhance the overall performance of classifiers trained with different models, provided the appropriate classifier is selected for the task and the training data are carefully selected. However, performance consistently diminished when classifiers were evaluated using dataset 3. This result is expected as dataset 3 places greater emphasis on the boundary region. Consequently, the data from dataset 1 or dataset 2, which are more general and lack detailed information around the boundary, failed to adequately train the algorithm to accurately identify this boundary. However, when the training data were concentrated around the boundary and subsequently the test was expanded to include a broader dataset, performance became comparable, regardless of whether datasets 1 or 2 were used.

The strong performance of KNN, DT, RF, and SVM-RBF classifiers suggests that the feature space could be divided into different non-overlapping regions, which could be considered by the trajectory planning algorithm. One possibility would be ranking the regions by feature stability and ensuring that the step ends in the geometric center of these regions. Additionally, to reduce the number of FN, the robot could be programmed to take action only if the last two or more steps were predicted to be unstable. However, this would result in slower reaction time to actual unstable steps, potentially leading to a fall.

Concerning the robot model, it is important to highlight that the PSV method opts for a detailed model over a simplified one for its multi-stage optimization. This choice is crucial because the datasets used contain configurations that deviate significantly from normal walking patterns and undergo substantial changes during recovery steps. Utilizing a simplified model, such as the inverse pendulum, would fail to accurately predict most outcomes in this scenario. Despite imposing restrictions on positions and velocities based on the theoretically recoverable limit of step capture, a considerable number of points remain unrecoverable. This observation implies that our approach is better at predicting step recoverability after major disruptions in walking patterns.

Future studies should leverage the trained classifiers to evaluate their effectiveness across various scenarios, including 3D movements in more sophisticated models. Investigating the impact of disparate models, sensor inaccuracies, and terrain inconsistencies on prediction accuracy using a real robot would validate the promising results observed in simulations. It is crucial to highlight the importance of integrating prediction and trajectory planning. This integration can be enhanced by incorporating various clusters within the feature space into trajectory planning, aligning them with stability predictions. This approach ensures a more comprehensive trajectory planning process, optimizing the robot’s performance in varying conditions. Moreover, since each prediction takes only a fraction of a millisecond, once it is determined that the current step is stable, the prediction could be used to plan future steps similarly to what the PSV algorithm achieves but in real-time.

## Figures and Tables

**Table 1:** Exoskeleton model parameters.

	Index		
	1 and 5	2 and 4	3
**mass (m)**	3.2kg	6.8kg	20kg
**length (l)**	0.4m	0.4m	0.625m
**Inertia (I)**	$0.93\;\mathrm{kg}\cdot m^{2}$	$1.08\;\mathrm{kg}\cdot m^{2}$	$2.22\;\mathrm{kg}\cdot m^{2}$
**distance (c)**	0.128m	0.163m	0.2m
$U_{\max}$	300N·m		

**Table 2:** Robotic model parameters.

	Index		
	1 and 5	2 and 4	3
**mass (m)**	0.254kg	0.780kg	3.861kg
**length (l)**	0.15m	0.15m	0.2095m
**Inertia (I)**	$4.34\;\mathrm{kg}\cdot {\mathrm{cm}}^{2}$	$8.74\;\mathrm{kg}\cdot {\mathrm{cm}}^{2}$	$141.48\;\mathrm{kg}\cdot {\mathrm{cm}}^{2}$
**distance (c)**	0.090m	0.085m	0.138m
$U_{\max}$	11.3N·m		

**Table 3:** Summary of results of data set I with balanced accuracy as the main metric.

Method	B. Acc.	ROC AUC	f1	Avg. Prec.	Prec.	Recall	Specificity	NPV
**NB**	0.9109	0.9109	0.6253	0.4506	0.4620	0.9674	0.8545	0.9951
**LogReg**	0.9229	0.9229	0.6875	0.5175	0.5383	0.8410	0.8946	0.9930
**KNN**	0.9369	0.9369	0.7096	0.5464	0.5584	0.9731	0.9006	0.9962
**SVM-L**	0.9226	0.9226	0.6796	0.5090	0.5273	0.9558	0.8893	0.9936
**SVM-RBF**	0.9569	0.9569	0.7649	0.6183	0.6227	0.9914	0.9224	0.9988
**DT**	0.9442	0.9442	0.7806	0.6352	0.6620	0.9510	0.9373	0.9933
**RF**	0.9506	0.9506	0.7861	0.6439	0.6634	0.9645	0.9368	0.9951
**AdaBoost**	0.9309	0.9309	0.7213	0.5580	0.5813	0.9501	0.9116	0.9930
**QDA**	0.9090	0.9090	0.6131	0.4385	0.4475	0.9731	0.8448	0.9959
**LDA**	0.9115	0.9115	0.6155	0.4417	0.4491	0.9779	0.8451	0.9966
**Ensemble**	0.9442	0.9442	0.7806	0.6352	0.6620	0.9510	0.9373	0.9933
**MLP**	0.9551	0.9551	0.7688	0.6227	0.6306	0.9846	0.9255	0.9979

**Table 4:** Summary of results of data set II with balanced accuracy as the main metric.

Method	B. Acc.	ROC AUC	f1	Avg. Prec.	Prec.	Recall	Specificity	NPV
**NB**	0.8659	0.8659	0.3649	0.2068	0.2308	0.8702	0.8615	0.9929
**LogReg**	0.8929	0.8929	0.4093	0.2439	0.2644	0.9062	0.8796	0.9949
**KNN**	0.9188	0.9188	0.4361	0.2716	0.2827	0.9531	0.8845	0.9975
**SVM-L**	0.8960	0.8960	0.4025	0.2404	0.2577	0.9182	0.8737	0.9956
**SVM-RBF**	0.9475	0.9475	0.6570	0.4772	0.5053	0.9389	0.9561	0.9970
**DT**	0.9239	0.9239	0.4739	0.3014	0.3162	0.9455	0.9024	0.9971
**RF**	0.9235	0.9235	0.4674	0.2964	0.3102	0.9476	0.8994	0.9972
**AdaBoost**	0.8918	0.8918	0.4557	0.2757	0.3077	0.8779	0.9057	0.9936
**QDA**	0.9007	0.9007	0.3604	0.3215	0.2217	0.9629	0.8386	0.9979
**LDA**	0.8798	0.8798	0.3642	0.2108	0.2279	0.9062	0.8534	0.9948
**Ensemble**	0.9475	0.9475	0.6570	0.4772	0.5053	0.9389	0.9561	0.9970
**MLP**	0.9435	0.9435	0.6293	0.4467	0.4738	0.9367	0.9503	0.9968

**Table 5:** Summary of results of data set III with balanced accuracy as the main metric.

Method	B. Acc.	ROC AUC	f1	Avg. Prec.	Prec.	Recall	Specificity	NPV
**NB**	0.6747	0.6747	0.6346	0.5688	0.6436	0.6259	0.7235	0.7080
**LogReg**	0.7178	0.7178	0.6934	0.6059	0.6689	0.7198	0.7158	0.7621
**KNN**	0.8148	0.8148	0.7960	0.7163	0.7784	0.8145	0.8150	0.8463
**SVM-L**	0.7176	0.7176	0.6934	0.6056	0.6684	0.7203	0.7149	0.7622
**SVM-RBF**	0.8329	0.8329	0.8156	0.7400	0.7999	0.8320	0.8339	0.8615
**DT**	0.8106	0.8106	0.7903	0.7142	0.7827	0.7979	0.8233	0.8363
**RF**	0.8377	0.8377	0.8199	0.7497	0.8143	0.8255	0.8498	0.8593
**AdaBoost**	0.7761	0.7761	0.7542	0.6700	0.7365	0.7728	0.7794	0.8113
**QDA**	0.7334	0.7334	0.7234	0.6132	0.6526	0.8115	0.6553	0.8134
**LDA**	0.7150	0.7150	0.6909	0.6029	0.6650	0.7190	0.7111	0.7603
**Ensemble**	0.8147	0.8147	0.7960	0.7163	0.7784	0.8145	0.8150	0.8463
**MLP**	0.8348	0.8348	0.8185	0.7390	0.7926	0.8463	0.8233	0.8704

**Table 6:** Out-of-set validation of data set I classifiers.

Method	TP 1	TN 1	FP 1	FN 1	TP 2	TN 2	FP 2	FN 2	TP 3	TN 3	FP 3	FN 3
**NB**	11.06	75.68	12.89	0.37	1.17	56.35	39.09	3.39	11.65	40.63	15.00	32.72
**LogReg**	10.88	79.23	9.33	0.56	4.22	57.76	37.69	0.33	41.83	6.42	49.20	2.55
**KNN**	11.13	79.76	8.80	0.31	4.36	34.80	60.64	0.19	43.31	3.64	51.98	1.07
**SVM_linear**	10.93	78.76	9.80	0.50	4.31	50.79	44.65	0.25	42.16	5.44	50.18	2.22
**SVM_RBF**	11.34	81.69	6.87	0.10	0.05	95.41	0.03	4.51	0.22	55.60	0.02	44.15
**DT**	10.88	83.01	5.55	0.56	3.11	71.39	24.05	1.45	28.16	27.43	28.20	16.22
**RF**	11.03	82.97	5.60	0.41	3.11	78.45	16.99	1.45	27.90	27.85	27.77	16.47
**AdaBoost**	10.87	80.74	7.83	0.57	1.83	77.10	18.34	2.73	22.65	28.37	27.25	21.73
**QDA**	11.13	74.82	13.74	0.31	0.33	94.32	1.12	4.23	4.48	52.20	3.42	39.89
**LDA**	11.18	74.84	13.72	0.25	3.19	75.19	20.26	1.37	33.17	24.69	30.93	11.20
**Ensemble**	10.88	83.01	5.55	0.56	3.11	71.39	24.05	1.45	28.16	27.43	28.20	16.22
**MLP**	11.26	81.97	6.60	0.18	3.66	85.01	10.43	0.89	39.12	19.78	35.84	5.26

**Table 7:** Out-of-set validation of data set II classifiers.

Method	TP 1	TN 1	FP 1	FN 1	TP 2	TN 2	FP 2	FN 2	TP 3	TN 3	FP 3	FN 3
**NB**	10.36	73.88	14.69	1.08	3.97	82.23	13.22	0.59	42.90	3.58	52.05	1.47
**LogReg**	11.33	15.84	72.73	0.11	4.13	83.95	11.49	0.43	43.82	9.73	45.89	0.55
**KNN**	11.27	55.04	33.52	0.16	4.34	84.42	11.02	0.21	43.93	9.91	45.71	0.45
**SVM_linear**	11.33	18.85	69.72	0.11	4.18	83.39	12.05	0.37	44.13	7.73	47.89	0.25
**SVM_RBF**	10.62	74.23	14.33	0.81	4.28	91.25	4.19	0.28	41.68	28.45	27.17	2.69
**DT**	10.80	74.49	14.07	0.64	4.31	86.12	9.32	0.25	43.29	10.54	45.09	1.09
**RF**	10.88	75.09	13.48	0.56	4.32	85.84	9.60	0.24	44.14	8.78	46.85	0.23
**AdaBoost**	11.03	67.69	20.88	0.41	4.00	86.44	9.00	0.56	44.29	2.67	52.96	0.08
**QDA**	8.60	78.20	10.36	2.83	4.39	80.04	15.41	0.17	44.28	3.12	52.50	0.09
**LDA**	10.88	61.63	26.93	0.56	4.13	81.45	13.99	0.43	44.25	3.96	51.66	0.13
**Ensemble**	10.62	74.23	14.33	0.81	4.28	91.25	4.19	0.28	41.68	28.45	27.17	2.69
**MLP**	10.98	50.46	38.11	0.46	4.27	90.70	4.74	0.29	42.48	25.73	29.89	1.89

**Table 8:** Out-of-set validation of data set III classifiers.

Method	TP 1	TN 1	FP 1	FN 1	TP 2	TN 2	FP 2	FN 2	TP 3	TN 3	FP 3	FN 3
**NB**	8.78	72.36	16.20	2.66	2.54	89.59	5.85	2.02	27.78	40.24	15.38	16.60
**LogReg**	10.38	14.47	74.10	1.05	2.78	91.14	4.30	1.77	31.94	39.82	15.81	12.43
**KNN**	10.38	35.79	52.77	1.05	3.33	84.85	10.60	1.23	36.14	45.34	10.29	8.23
**SVM_linear**	10.38	15.46	73.10	1.05	2.79	91.39	4.06	1.76	31.97	39.77	15.86	12.41
**SVM_RBF**	7.88	80.88	7.68	3.56	2.78	94.47	0.97	1.77	36.92	46.39	9.24	7.46
**DT**	11.05	7.99	80.57	0.38	3.17	84.93	10.52	1.39	35.41	45.80	9.83	8.97
**RF**	10.90	24.28	64.28	0.54	3.40	85.43	10.01	1.16	36.63	47.27	8.35	7.74
**AdaBoost**	11.26	5.78	82.78	0.18	3.14	85.57	9.87	1.42	34.29	43.35	12.27	10.08
**QDA**	4.63	83.60	4.96	6.80	3.10	90.09	5.35	1.46	36.01	36.45	19.17	8.36
**LDA**	10.41	17.59	70.97	1.03	2.78	91.69	3.75	1.78	31.90	39.56	16.07	12.47
**Ensemble**	10.38	35.79	52.77	1.05	3.33	84.85	10.60	1.23	36.14	45.34	10.29	8.23
**MLP**	10.19	78.49	10.08	1.25	3.03	94.52	0.92	1.53	37.55	45.80	9.83	6.82

## Data Availability

The data presented in this study are available on request from the authors.
